# Supplementary material for: Isotopic evidence for geographic heterogeneity in Ancient Greek military forces
Source: PLoS One. 2021 May 12;16(5):e0248803. doi: 10.1371/journal.pone.0248803 (PMC8115791; doi:10.1371/journal.pone.0248803)
Supplement: S1 Table — (PDF) [file pone.0248803.s001.pdf]

Archaeological Specimens stored at the Parco Archeologico di Himera, Sicily, Italy
W1656
W2574
W1480
W1788
W5209
W2463
W2472
W1896
W6049
W1901
W6083
W2485
W6111
W4324
W2301
W3612
W3702
W2468
W2831
W3182
W0303
W1838
W6112
W6022
W2499
W336
W482
W403
W276
W396
W494
W428
W464
W503
W461
W429
W577
W462
W463
W576
W737
W808
W810
W696
W814
W809
W701
W702
W699
W812
W706
W704
W705
W807
W703
W653
W698
W811
W650
W1783
W1781

W1770
W1777
W1773
W1779
W1771
W1774
W2588
W2587
W2589
W2590
W2737
W2738
W2739
W2825
W2764
W4378
W4376
W4380
W4674
W4666
W4680
W4651
W4670
W4689
W4684
W4687
Archaeological specimens stored at the Bioarchaeology and Biochemistry Laboratory, University of Georgia, Athens, GA, USA
H-W1993
H-W1114-P
HMFCAP
H-W3704-D
H-W704-H
H-W-3030-H
